# Indicators of antioxidant protection of blood in necrotizing ulcerative gingivitis in experimental animals

**DOI:** 10.25122/jml-2020-0149

**Published:** 2021

**Authors:** Yuliia Hafizivna Kilmukhametova, Viktor Markiyanovich Batig, Marianna Alexandrovna Ostafiichuk, Olha Mykhaylivna Tokar, Tatiana Anatoliyivna Glushchenko, Iryna Viktorivna Batih, Michael Ivanovich Sheremet

**Affiliations:** 1. Department of Therapeutic Dentistry, Higher State Educational Institution of Ukraine “Bukovinian State Medical University”, Chernivtsi, Ukraine; 2. Department of Pediatric Dentistry, Higher State Educational Institution of Ukraine “Bukovinian State Medical University”, Chernivtsi, Ukraine; 3. Surgery Department No.1, Higher State Educational Institution of Ukraine “Bukovinian State Medical University”, Chernivtsi, Ukraine

**Keywords:** antioxidants, free radicals, periodontitis, simulated necrotizing ulcerative gingivitis, experimental animals, MDA, LPO, CA, API, API – Antioxidant-Prooxidant Index, CA – Catalase, LPO – Lipid Peroxidation, MDA – Malonic Dialdehyde

## Abstract

This article highlights the results of a study of blood parameters in animals with simulated necrotizing ulcerative gingivitis and compares them, under the same conditions, with animals that received local treatment with a developed complex of antioxidant drugs. Following the work tasks, the nature of changes in the state of the antioxidant - prooxidant system and their influence on quantitative and functional indicators of markers of inflammatory intensity was analyzed and investigated during the pathological process in the background and without treatment with a developed complex. This work shows the changes of malonic dialdehyde concentration as an indicator of lipid peroxidation intensity in experimental animals, the level of catalase activity in the blood of animals, and antioxidant-prooxidant balance in the dynamics of necrotizing ulcerative gingivitis.

## Introduction

Periodontal disease is a chronic inflammatory process in the periodontal tissues caused by an imbalance between oral biofilms and the patient’s immune response. There is a possibility of a loss of tissues supporting the tooth [1–3]. Periodontitis is one of the most prevalent diseases of the oral cavity, caused by bacterial plaque microorganisms and influenced by different factors such as somatic diseases, oral hygiene, age, sex, and bad habits [4, 5].

Periodontal disease has been linked to the excessive presence of free radicals caused by oxidative stress or antioxidant deficiency [1, 6, 7]. In the early progression of periodontal disease, there is a marked oxidative process with increased levels of reactive oxygen and nitrogen species, especially in periodontitis. This process can lead to an imbalance in the body response, with concomitant changes in biomolecules, especially lipids, proteins, and nucleic acids, resulting in periodontal tissue damage [8]. The antioxidant defense system can inhibit and/or reduce the damage caused by the deleterious action of free radicals or non-radical reactive species [9]. Some antioxidant sources found in nature (foods, teas, vitamins, minerals, among others) are currently used in various auxiliary treatments for cardiovascular diseases, pulmonary diseases, aging, and atherosclerosis [10, 11]. As most of those somatic diseases have proved physiologic links with periodontal diseases, it was assumed that treatment with antioxidants could lead to a positive result in periodontal diseases. Some literature studies suggest that supplementation with antioxidant components may help to reduce periodontal damage and its systemic effects when compared to the usual treatment [12].

Antioxidant therapy is emerging as a promising new paradigm as prophylactic and therapeutic agents [13]. The reduction of molecular oxygen to hydrogen peroxide is accompanied by a large free energy release that can give rise to free radicals and reactive oxygen species [14]. They play a crucial role in normal physiological processes like response to growth factors, immune response, and apoptotic elimination of damaged cells. At the same time, they may also represent an important pathogenic mechanism for tissue damage and diseases associated with phagocyte infiltration when generated during respiratory burst [15]. Antioxidants are those substances that will significantly delay or inhibit oxidation of that substrate when present at low concentrations compared to those of an oxidizable substrate [16]. Hence, antioxidants are essential to counteract the damage caused by free radicals. Evidence is emerging that increased free radicals in the pathogenesis of periodontitis enhance tissue destruction [17, 18]. 

The purpose of the work was to study the indicators of antioxidant protection of blood in animals with simulated necrotizing ulcerative gingivitis and compare them, under the same conditions, with animals that received a local treatment for this pathology with a developed complex of antioxidant drugs.

## Material and Methods

Experimental laboratory studies on animals have been performed following the principles of bioethics and the basic provisions of the Good Clinical Practice (1996), the Council of Europe Convention on Human Rights and Biomedicine (04.04.1997), the Helsinki Declaration of the World Medical Association on the ethical principles of scientific medical research with human participation (1964–2013), an order of the Ministry of Health of Ukraine no. 690 from 23.09.2009 and no. 616 from 03.08.2012. The Commission on Biomedical Ethics of the Bukovinian State Medical University of the Ministry of Health of Ukraine (Chernivtsi) (Protocol no. 7, April 20, 2017) did not reveal any violations of moral and ethical norms during the research work.

Experimental studies were conducted based on the vivarium of the L’viv State Research Institute of Veterinary Drugs and Feed Additives. In the course of research work, experimental studies were conducted on 18 male rabbits weighing 2–2.5 kg. An experimental model of necrotizing ulcerative gingivitis was obtained in animals by chemical burns.

According to the conditions of the experiment, all animals were divided into three groups:

1. Intact animals (6 rabbits);
2. Control group – animals of this group were not treated, the necrotizing ulcerative lesions on the mucous membrane of the alveolar sprout of the upper jaw healed independently (6 rabbits);
3. Experimental group – in these animals, local treatment with a complex of antioxidant drugs (ointment Tiotriazoline, ointment Zinc Oxide, and Chlorhexidine digluconate) was used starting from the day of necrotizing ulcerative gingivitis modeling, during all observation periods (6 rabbits) [19, 20, 21].

Experimental drugs in an approximate dose of 200 mg were applied to the damaged area of the gums 2 times a day, 2 hours after feeding the animals. Melted paraffin was used to fix the drugs on the wound surface.

Since the nature of the course of experimental necrotizing ulcerative gingivitis was studied during the work, the key stages of observation were the key stages of healing: the 3**rd** day – the peak of the inflammatory process, the 5**th** day – completion of necrolysis on the ulcer surface; the 7**th** day – intensive, regenerative processes; the tenth day – completion of the pathological process with epithelialization of the damaged area. At this time, under ether anesthesia, blood was drawn from the ear vein of each experimental animal [22].

Observation of the condition of the damaged mucous membrane of the frontal maxillary alveolar sprout was performed daily, photographing the wound according to the scheme of the experiment (on the 3**rd**, 5**th**, 7**th**, and 10**th** days). The criteria for assessing the effectiveness of local treatment were the timing of elimination of perifocal inflammation, redness, infiltration of the edges of the lesion, cleaning of the necrotic tissue surface, the beginning of marginal epithelialization, and the time of its completion.

Mathematical and statistical processing of the obtained research results was performed using a personal computer with the appropriate software package (StatSoft Statistica, v8), which is recommended for this processing method. Given that all the studied data were variation series with a statistical set of normal (symmetrical) distribution when analyzing the statistical characteristics of individual groups, generally accepted indicators of descriptive statistics with the definition of values were used: mean (M) ± standard error (m). Comparison of mean values in different groups was performed using the classical parametric t-test. When comparing the results, we used the estimation of differences by the adequate method for small samples, using the table of Student’s criterion. The differences were considered significant at p <0.05. The density of the relationship between the state of the antioxidant-prooxidant ratio and markers of the intensity of the inflammatory process was studied using correlation analysis to determine the correlation coefficient (r).

## Results and Discussions

### Dynamics of malonic dialdehyde concentration as an indicator of lipid peroxidation intensity in experimental animals

An in-depth study of the pathogenesis of necrotizing ulcerative gingivitis has indicated an intensification of free radical oxidation reactions of organic structures, which plays a marginal role in expanding the lesion by so-called “secondary alteration” and the persistence of the pathological process. One of the adverse effects of lipid peroxidation (LPO) due to free radical breakdown of polyunsaturated fatty acids is considered the formation of malonic dialdehyde (MDA). With its participation as a “crosslinking” agent, alkalis with amino groups of proteins are formed, as a result of which insoluble lipid-protein complexes (lipofuscins) appear, and the barrier and matrix function of cell membranes is disturbed [23, 24].

The concentration of MDA in the serum reflects the activity of LPO processes in the body and serves as an indicator of the magnitude of endogenous intoxication. As a rule, its high content corresponds to the severe degree of the disease. Thus, the increased content of MDA in the serum is found in myocardial infarction, acute respiratory and hepatic failure, acute inflammation and sepsis, traumatic brain injury, and other diseases [25–27]. That is why MDA is considered an objective marker of the intensity of LPO processes and is studied in the study of the mechanisms of development of various pathological processes and the development of methods for their correction.

**Figure 1.**
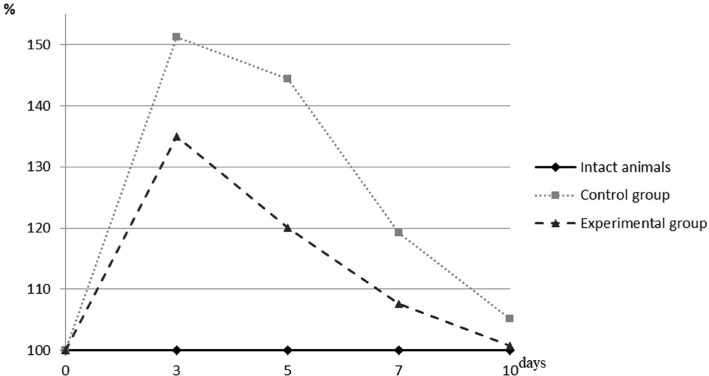
The percentage of MDA in the blood of animals of different experimental groups to the rate of intact group.

Given the above, this study aimed to investigate changes in the concentration of MDA in the blood of experimental animals with experimental necrotizing ulcerative gingivitis and the possibility of influencing their course with the developed complex of antioxidant drugs applied in the form of a periodontal dressing. The obtained quantitative results are presented in Table 1 and Figure 1.

**Table 1:** The concentration of MDA in the blood of experimental animals on the background of treatment and without treatment (M ± m, n = 6).

Groups	Terms of observation; obtained results, μmol/l				
	Intact animals	3**rd** day	5**th** day	7**th** day	10**th** day
C	3.64±0.10	5.58±0.16 p1<0.001	5.33±0,15 p1<0.001	4.40±0.13 p1<0.001	3.88±0.17 p1=0.216
E	3.64±0.10	4.98±0.19 p1<0.001 p2=0.040	4.43±0.15 p1<0.001 p2=0.002	3.97±0.14 p1=0.078 p2=0.037	3.72±0.11 p1=0.636 p2=0.441

p1 – reliability for intact animals;

p2 – reliability in relation to the control group of animals.

As can be seen, the nature of the change in the concentration of MDA in both experimental groups of animals was the same but differed in its magnitude of growth and rate of normalization. In animals of the untreated control group, on the 3**rd** day, there was a maximum increase in the concentration of MDA (51.21%), which significantly (p <0.001) exceeded the same value in intact animals. Subsequently, a gradual decrease in the studied value was determined. However, on the 5**th** and 7**th** day, its value still differed significantly from the level of intact animals and was 44.44% (p <0.001) and 119.24% (p<0.001), respectively. It should be noted that there was a slight difference between the 3**rd** and 5**th** days and a much more intense fall between the 5**th** and 7**th** days. At the end of the observation, the value of the concentration of MDA in the control group did not differ significantly from its value in intact animals and amounted to 105.14% at a p-value of 0.216.

The maximum increase in the concentration of MDA in the blood of animals of the experimental group was also seen in the first observation period when its value exceeded the data of intact animals by 34.95% (p <0.001). In the next two terms, there was a rapid decrease in this group regarding the content of MDA: while on the 5**th** day, its value still significantly exceeded the level of the intact group by 20.05% (p <0.001), on the 7**th** day, it exceeded only by 7.98% at a p-value of 0.078, which indicated a statistically insignificant difference. At the end of the experiment, its concentration did not differ from that of intact animals and amounted to 100.81% at a p-value of 0.636.

### The level of catalase activity in the blood of animals of different groups

The damaging effects of free radicals, which are constantly formed in the body, are counteracted by a multi-stage antioxidant defense system. One of its components is the enzymatic link, a representative of which is catalase (CA) – an enzyme that decomposes hydrogen peroxide formed during biological oxidation into water and molecular oxygen and oxidizes in the presence of hydrogen peroxide low-molecular-weight alcohols and nitrites and thus participates in the process of cellular respiration. Catalase is one of the fastest enzymes; one molecule of catalase can convert several million molecules of hydrogen peroxide into water and oxygen per second and is found in almost all organisms [28, 29].

**Figure 2.**
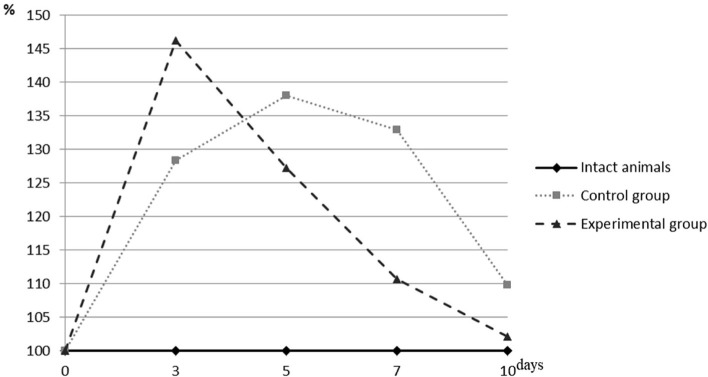
Percentage of CA in the blood of animals of different experimental groups to the rate of intact animals.

Since the main function of catalase is to destroy toxic hydrogen peroxide formed during various oxidative processes in the body, this study aimed to determine the changes in its activity in the blood of animals with simulated necrotizing ulcerative gingivitis on the background of treatment and without treatment with a developed antioxidant complex. The obtained results are presented in Table 2 and Figure 2.

**Table 2:** Catalase activity in the blood of experimental animals on the background of treatment and without treatment (M ± m, n = 6).

Groups	Terms of observation; obtained results, μmol/l				
	Intact animals	3**rd** day	5**th** day	7**th** day	10**th** day
C	16.64±0.38	21.36±0.39 p1<0.001	22.97±0.42 p1<0.001	22.11±0.22 p1<0.001	18.26±0.24 p1=0.012
E	16.64±0.38	24.33±0.29 p1<0.001 p2<0.001	21.17±0.37 p1<0.001 p2=0.009	18.42±0.21 p1<0.001 p2<0.001	17.00±0.40 p1=0.562 p2=0.022

p1 – reliability for intact animals; p2 – reliability in relation to the control group of animals.

As can be seen, there were changes in the activity of CA when comparing the data obtained in each of the experimental groups. In the initial period, there was an increasing tendency of its level in animals of the control group. On the 3**rd** day, the enzymatic activity increased by 28.36% (p <0.001) relative to the level of intact animals and continued its increase with the achievement of the maximum value throughout the observation period of this group on the 5**th** day with a result of 38.04% (p <0.001) that significantly outweighed the level of the intact group. By the 7**th** day, the activity of this enzyme changed slightly and continued to significantly exceed the level of intact animals by 32.87% (p <0.001). Only at the final stage of observation was its sharp decline determined, reaching the value seen in the intact animals (109.73%). However, this value of p = 0.012 continued to differ significantly from the established physiological data.

The maximum increase in the enzymatic activity of catalase in the experimental group was detected in the first term of the study (3**rd** day), with a significant excess in intact animals by 46.21% (p <0.001). In the next two terms, its value decreased significantly: on the 5**th** day, the value of intact animals was 27.22% (p <0.001), and on the 7**th** day it was 10.69% (p<0.001). The further decline continued. At the end of the observation (the 10**th** day), the established value was already insignificantly different from the level of intact animals and was equal to 102.16% at a p-value of 0.562.

### Antioxidant-prooxidant balance in the dynamics of necrotizing ulcerative gingivitis

Under physiological conditions, the processes of free radical formation occur permanently and under the control of a multi-stage system of antioxidant protection. As a result, a stable antioxidant-prooxidant balance is formed in the tissues. For its complex characterization, an integrated indicator such as an antioxidant-prooxidant index (API) is often used in the scientific literature. It is determined by the ratio of the activity of the antioxidant enzyme catalase to the number of secondary products of lipoperoxidation – malonic dialdehyde [30–32].

**Figure 3.**
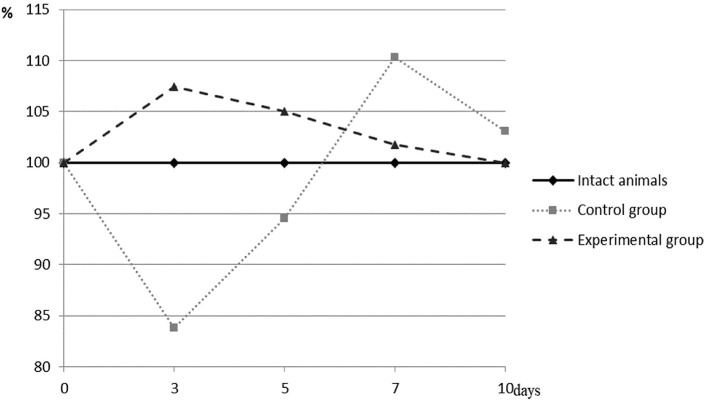
Percentage of blood API of animals of different experimental groups to the rate of intact animals.

By using the indicators obtained in the previously described studies, the value of API for each group of experimental animals was calculated at all observation times and is depicted in Table 3 and Figure 3 to determine the violations of this balance and establish their causes (activation or suppression of both one and its antagonistic system).

**Table 3:** The value of blood API of experimental animals in the dynamics of necrotizing ulcerative gingivitis (M ± m, n = 6).

Groups	Terms of observation; obtained results, μmol/l				
	Intact animals	3**rd** day	5**th** day	7**th** day	10**th** day
C	4.57±0.03	3.83±0.04 p1<0.001	4.32±0.05 p1<0.001	5.04±0.09 p1<0.001	4.71±0.15 p1=0.228
E	4.57±0.03	4.91±0.13 p1=0.004 p2<0.001	4.80±0.13 p1=0.006 p2<0.001	4.65±0,10 p1=0.327 p2=0.018	4.57±0.03 p1=1 p2=0.388

p1 – reliability for intact animals; p2 – reliability in relation to the control group of animals.

In animals of the control group, there was a sharp, significant decrease in the value of API with a drop to 83.81% (p <0.001) at the beginning of the study (the 3**rd** day). The next period (the 5**th** day) showed an increase in the value of intact animals and was already 94.52% (p <0.001). The shift towards the antioxidant system was detected on the 7**th** day and was 110.28% (p <0.001) compared to healthy animals. After that, there was a tendency to normalize and decrease the value of the studied index to 103.06% at a p-value of 0.228 on the 10**th** day of the experiment.

By its nature, the dynamics of API in animals of the experimental group, which received the appropriate local treatment with the developed complex of antioxidants according to the conditions of the experiment, was notably different from the dynamics found in the control group. On the 3**rd** day, there was already a significant increase in the value of this indicator to 107.44% (p <0.001) compared to the level of intact animals. In all subsequent terms, a gradual decrease in the value of API was determined. If its value was equal to 105.03% on the 5**th** day with a significant difference to the physiological level at a p-value of 0.006, on the 7**th** day, this difference was statistically insignificant, and the value itself was 101.75% (p = 0.327) in case of intact animals. At the end of the experiment (10**th** day), the API data completely coincided with the data type of intact animals.

Thus, it is possible to state a noticeable imbalance in the body with a shift in the prooxidant direction in the initial stages of necrotizing ulcerative gingivitis due to the intensive formation of free radical compounds and activation of lipoperoxidation and inhibition of antioxidant protection. Signs of normalization are observed only on the 10**th** day of the pathological process. Local treatment with the developed complex of antioxidants reduces the level of LPO in the damaged tissues, namely the load on the antioxidant protection system and avoiding its overload. As a result, it is possible to achieve a physiological balance of these two systems on the 7**th** day of the disease.

### Visual observation

In animals of both experimental groups, there was a clinical picture that is characteristic of the acute inflammatory process in the initial period of observation (3**rd** day). It was most pronounced in animals of the control group. The mucosa around the simulated ulcer was intensely hyperemic, swollen; the edges became roller-shaped, which visually increased the area of damage. The very surface of the ulcer was completely covered with a white plaque, which was firmly welded to the underlying tissues and was challenging to remove with a spatula. In the animals of the experimental group, on the 3**rd** day, it was already possible to notice less pronounced hyperemia and edema of the surrounding tissues. The edges of the ulcer were contoured, and its surface was filled with a white plaque, which was easily removed and only partially covered with crusts on the side in the area of lip tissue, which could be removed with a spatula.

On the 5**th** day, it was already possible to notice significant differences in the dynamics of healing of the simulated ulcers of the oral mucosa. The control group animals still had signs of acute inflammatory process with hyperemia and tissue edema, and the entire surface was covered with necrotic masses, which were difficult to remove from the wound surface.

On the 5**th** day, hyperemia and exudate phenomena in the surrounding tissues were no longer detected in animals of the experimental group. A significant surface of the wound was clear of necrotic masses and began to show signs of epithelial tissue growth from the ulcer’s edges, and only small areas continued to be covered with thin light brown color. In the control group, day 7 was characterized by the absence of acute inflammatory phenomena. At this time, the ulcer surface is almost completely cleared of necrotic masses and filled with granulation tissue, and the remnants of necrosis can be found only in its central area. The initial signs of epithelial tissue growth appear near the edges of the intact mucous membrane.

At this time (7**th** day), in the experimental group, there was a sharp decrease in the area of the ulcer due to the active epithelialization processes passing from the edges of the defect of the mucous membrane. The central non-epithelialized part is filled with pale pink granulation tissue. Completion of observation (10**th** day) in the control group was characterized by incomplete epithelialization of the surface of the simulated ulcer. All animals of this group had a small area that was not covered with epithelium in the central part. In contrast, in the animals of the experimental group, the entire damaged area of the mucosa was covered with epithelium on the 10**th** day, which differed from the surrounding only by lighter pigmentation.

## Conclusions

In experimental necrotizing ulcerative gingivitis, there is an activation of LPO processes with an increase of its secondary products in the blood by 1.53 times on the 3**rd** day. As a result of this stress, inhibition of the enzymatic activity of antioxidant protection occurs. As a result, there is an imbalance in the body with a shift to the prooxidant side, which lasts up to the 5**th** day with a gradual shift in the antioxidant direction, which persists until the 10**th** day of observation.

Local application of the developed complex of antioxidants reduces the content of lipoperoxidation products, particularly the concentration of MDA, which avoids overstrain and depletion of antioxidant defense systems, resulting in a shift of balance in the antioxidant direction and its normalization on the 7**th** day. Additionally, it allows correcting a course of inflammatory reaction that damaged the mucous membrane of an oral cavity. As a result, a moderate growth of the studied markers was noted in the initial phase, and it was smaller compared to the control group. Also, their full normalization was seen on the 7**th** day of observation.

## Acknowledgment

### Conflict of interest

The authors declare that there is no conflict of interest.
